# Evaluation of needle movement effect on root canal irrigation using a computational fluid dynamics model

**DOI:** 10.1186/s12938-019-0679-5

**Published:** 2019-05-06

**Authors:** Shanshan Hu, Lunliang Duan, Qianbing Wan, Jian Wang

**Affiliations:** 1 State Key Laboratory of Oral Diseases, National Clinical Research Center for Oral Diseases, West China Hospital of Stomatology, Sichuan University, Chengdu, China; 2 Department of Bridge Engineering, Southwest Jiaotong University, Chengdu, 610031 China; 3 Department of Prosthodontics, West China Hospital of Stomatology, Sichuan University, Chengdu, 610041 China

**Keywords:** Computational fluid dynamics, Root canal treatment, Irrigation, Needle movement, Periapical extrusion

## Abstract

**Background:**

Irrigation is considered to be a critical part of root canal treatment. However, little is known about the effect of needle movement on the irrigation process. Therefore, this study aimed to investigate the influence of the syringe and needle movement on root canal irrigation using a three-dimensional computational fluid dynamics (CFD) numerical model.

**Methods:**

The CFD codes Flow-3D was adopted to simulate the root canal irrigation process with the syringe and needle moving up and down in motions at different amplitudes and frequencies. One stationary needle was adopted to allow comparison with the needles in up-and-down motions. Six cases where the needles were moving up and down with different amplitudes and frequencies were used to investigate the relationships between the motion of needle and irrigation efficacy.

**Results:**

The stationary needle gained relatively higher flow velocity and apical pressure all through the irrigation process, while the needles in constant up-and-down motions exhibited lower mean flow velocity and apical pressure. The larger the amplitude, the less mean flow velocity and apical pressure were developed. In addition, the needles moving with different frequencies were similar in the terms of irrigant replacement and apical pressure.

**Conclusions:**

To avoid periapical extrusion accidents while obtaining adequate irrigant replacement, the needle should be moving up and down with a moderate amplitude during manual root canal irrigation; and the motion frequency was not highly relevant in terms of the irrigation efficiency.

**Electronic supplementary material:**

The online version of this article (10.1186/s12938-019-0679-5) contains supplementary material, which is available to authorized users.

## Background

Irrigation is considered to be a critical part of root canal treatment because it allows for the debridement and disinfection of the root canal, which cannot be achieved by root canal instrumentation alone [1, 2]. In addition, several studies have demonstrated that irrigation of the apical third of the root canal is extremely challenging not only because of the complexity of the root canal structure [3, 4], but also due to the fact that the microorganisms in this area principally communicate with the host and may cause periapical inflammation [5].

Among all the irrigation techniques, conventional syringe irrigation is still considered as the most widely used method [6, 7], with the irrigant being delivered into the root canal by a syringe and needle, either passively or with agitation. The latter is achieved by moving the needle up and down the canal space. Moreover, using this conventional irrigation method, the depth of needle penetration and the volume of irrigant could be comparatively better controlled [8, 9]. Many factors have been shown to be closely related to efficient syringe irrigation. For instance, high flow rates [10], closer approach of the needle to the apex [10], and larger irrigation volume [11]. However, since the irrigant is being injected directly under positive pressure into the canal, these measures might potentially pose a threat to a well-known complication: periapical extrusion of irrigant [12], which may result in severe tissue damage and postoperative pain [3]. Moreover, it would be difficult to standardize a limit for a safe irrigation protocol since irrigant extrusion in vivo is a multifactorial process and it is challenging to build a clinically relevant model to validate various factors.

Recently, by means of mathematical modeling and computer simulation, computational fluid dynamics (CFD) has been adopted to simulate the flow pattern developed within the root canal [13–17]. One outstanding advantage of CFD modeling is the visualization of the flow pattern developed within the root canal that experimental measurements are difficult to perform. Moreover, in contrast to experiments, various flow characteristics such as flow velocity and pressure could be easily obtained by CFD modeling, especially in microscale flow situations like the flow developed in the apical part of root canal [18]. In addition, the reliability of CFD root canal model has also been validated by various studies by means of high-speed imaging and particle image velocimetry analysis [18, 19]. Therefore, CFD might be considered as a promising technique to investigate the efficiency of syringe irrigation and the potential risk of irrigant extrusion. However, in almost all CFD analyses, the syringes and needles remain stationary and it has been pointed out that the needle should be kept in motion during manual irrigation to facilitate decontamination and reduce the possibility of irrigant extrusion [20, 21].

Presently, little is known about the effect of needle movement on the irrigation process and there is limited evidence for the recommendation of a safe yet effective irrigation protocol during root canal treatment. As a result, the present study established a three-dimensional CFD model to simulate the root canal irrigation process with the syringe and needle moving up and down in motions at different amplitudes and frequencies. The aim of this study was to evaluate the relationships between irrigation efficacy and the amplitudes and frequencies of the up-and-down motions. Moreover, the potential for periapical extrusion under different amplitudes and frequencies was investigated and the optimal irrigation strategy was also explored.

## Methods

### The geometry construction for a CFD irrigation model

**Fig. 1:**
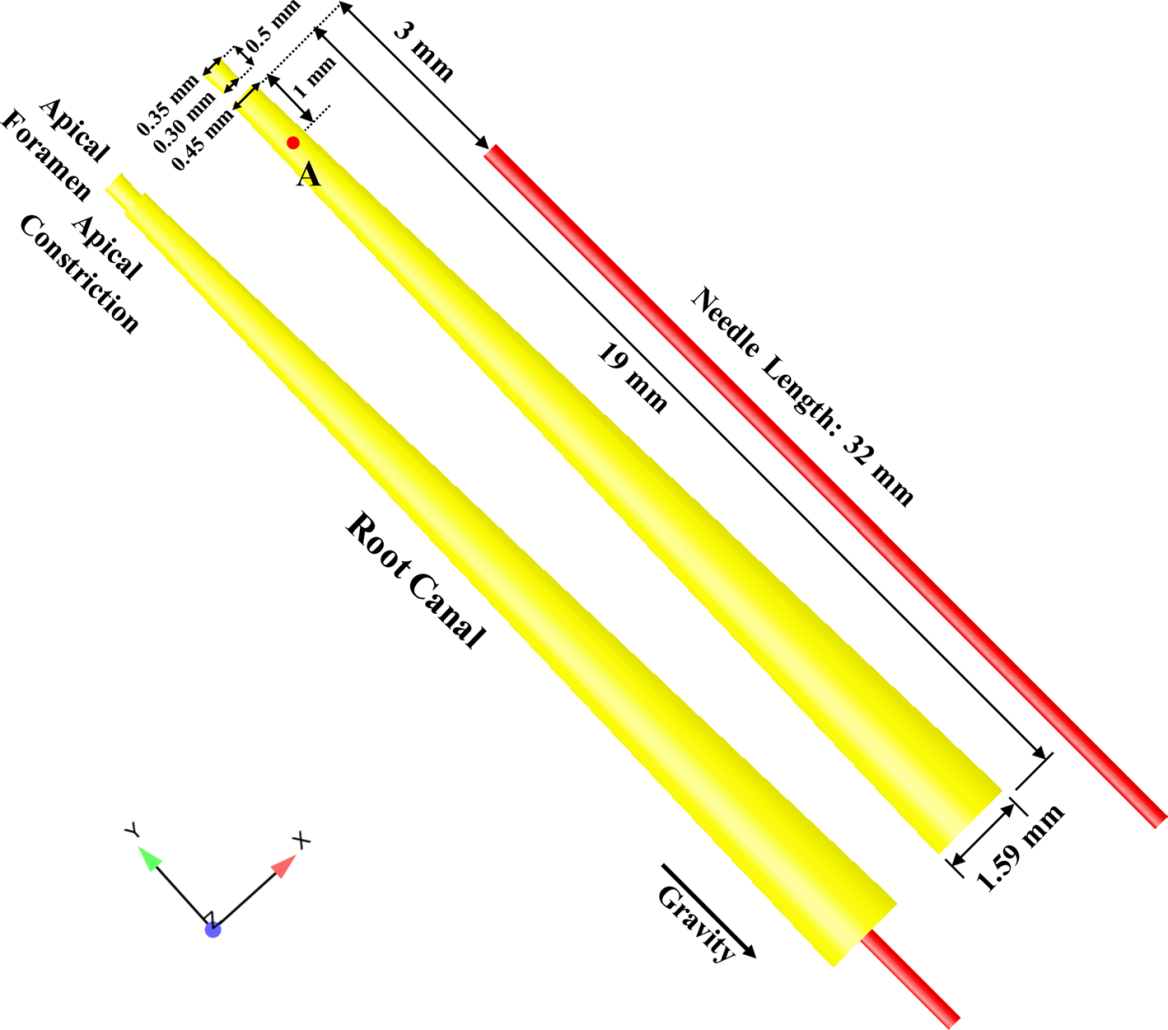
Sketch of the root canal model in the present study

The geometry of the root canal and the needle was created according to a previous study [13] with Auto CAD 2012 software (AutoDesk, San Rafael, CA, USA) (Fig. 1). A 30-G flat needle was selected in the present study and the external diameter, internal diameter, and length of the needle were 320 µm, 196 µm, and 31 mm, respectively. All the needles were initially placed at 3.5 mm above the apical foramen to facilitate standardization among different cases. In addition, point A (1.5 mm from the apical foramen) was selected [14, 15] to analyze the variations of flow velocity under different cases (Fig. 1). In this study, 1% solution of sodium hypochlorite (NaOCl), with a density of 1.04 g/cm^3^, and a viscosity of 0.986 × 10^−3^ Pa s [22], was adopted and assumed to be an incompressible and homogeneous Newtonian fluid [23]. The inlet flow velocity of the needle was set to be 8.6 m/s, which was consistent with an irrigant flow rate of 0.26 ml/s through a 30-G flat needle [24].

### The governing equations

According to Sinaiski [25], the general condition for the beginning of turbulence has been established by Reynolds number (Re), which could be calculated by the formula Re = *ρvD*/*µ* (*ρ* was the fluid density, *v* was the fluid velocity, *D* was the domain diameter, and *µ* denoted the fluid viscosity). In the present study, *ρ* = 1.04 g/cm^3^, *µ *= 0.986 × 10^−3^ Pa s, *v* = 4.3 m/s (the mean velocity, which was half of the inlet flow velocity of the needle), D ranged from 0.63 mm to 0.81 mm for the two extreme positions of the needle tip (at 3.5 mm and 6.5 mm from the apex). Therefore, the Reynolds number in the studied case ranged from 2857 to 3674 depending on the position of the needle tip. In addition, the critical value of Re (Re_cr_) for a pipe flow is approximately equal to 2800. Namely, the flow remains laminar until the Re stays below Re_cr_, whereas at Re > Re_cr_, the flow becomes turbulent [25]. Therefore, the flow motions in the present study were modeled by solving the Reynolds-Averaged Navier–Stokes (RANS) equation and the *k*–*ε* turbulence model [26]. Due to the adoption of the FAVOR mesh processing technique in the Flow-3D codes, the area fraction and volume fraction were introduced in the governing equations. Therefore, the governing equations for flow simulations in the present study were as follows:

1 $$ \frac{\partial }{\partial x}(u_{x} A_{x} ) + \frac{\partial }{\partial y}(u_{y} A_{y} ) + \frac{\partial }{\partial z}(u_{z} A_{z} ) = 0, $$

2 $$ \frac{{\partial u_{x} }}{\partial t} + \frac{1}{{V_{F} }}\left\{ {u_{x} A_{x} \frac{{\partial u_{x} }}{\partial x} + u_{y} A_{y} \frac{{\partial u_{x} }}{\partial y} + u_{z} A_{z} \frac{{\partial u_{x} }}{\partial z}} \right\} = - \frac{1}{\rho }\frac{\partial p}{\partial x} + G_{x} + f_{x}, $$

3 $$ \frac{{\partial u_{y} }}{\partial t} + \frac{1}{{V_{F} }}\left\{ {u_{x} A_{x} \frac{{\partial u_{y} }}{\partial x} + u_{y} A_{y} \frac{{\partial u_{y} }}{\partial y} + u_{z} A_{z} \frac{{\partial u_{y} }}{\partial z}} \right\} = - \frac{1}{\rho }\frac{\partial p}{\partial y} + G_{y} + f_{y}, $$

4 $$ \frac{{\partial u_{z} }}{\partial t} + \frac{1}{{V_{F} }}\left\{ {u_{x} A_{x} \frac{{\partial u_{z} }}{\partial x} + u_{y} A_{y} \frac{{\partial u_{z} }}{\partial y} + u_{z} A_{z} \frac{{\partial u_{z} }}{\partial z}} \right\} = - \frac{1}{\rho }\frac{\partial p}{\partial z} + G_{z} + f_{z}, $$

where *x*, *y*, *z* were the coordinates; *ρ* was the fluid density; *t* represented the time; *u*_x_, *u*_y_, *u*_z_ denoted the fluid velocity in the *x*-, *y*-, *z*- direction, respectively; *A*_*x*_, *A*_*y*_, *A*_*z*_ signified the area fraction in the *x*-, *y*-, *z*- direction; *V*_F_ meant the volume fraction; *G*_*x*_, *G*_*y*_, *G*_*z*_ were the gravitational acceleration in the *x*-, *y*-, *z*- direction, respectively; and *f*_*x*_, *f*_*y*_, *f*_*z*_ denoted the viscous force due to the acceleration in the *x*-, *y*-, *z*- direction, respectively.

The standard *k*–*ε* turbulence model was adopted in this study to achieve the closure of the governing equations. Therefore, the *k*–*ε* equations after the introduction of the area fraction and the volume fraction could be expressed as

5 $$ \frac{{\partial k_{\text{T}} }}{\partial t} + \frac{1}{{V_{F} }}\left( {u_{x} A_{x} \frac{{\partial k_{\text{T}} }}{\partial x} + u_{x} A_{x} \frac{{\partial k_{\text{T}} }}{\partial x} + u_{x} A_{x} \frac{{\partial k_{\text{T}} }}{\partial x}} \right) = P_{\text{T}} + G_{\text{T}} + D_{\text{T}} - \varepsilon_{\text{T}}, $$

6 $$ \frac{{\partial \varepsilon_{\text{T}} }}{\partial t} + \frac{1}{{V_{F} }}\left( {u_{x} A_{x} \frac{{\partial \varepsilon_{\text{T}} }}{\partial x} + u_{y} A_{y} \frac{{\partial \varepsilon_{\text{T}} }}{\partial x} + u_{z} A_{z} \frac{{\partial \varepsilon_{\text{T}} }}{\partial x}} \right) = \frac{{C_{1} \varepsilon_{T} }}{{k_{T} }}\left( {P_{\text{T}} + C_{3} G_{\text{T}} } \right) + D_{\varepsilon } - C_{2} \frac{{\varepsilon_{\text{T}} }}{{k_{\text{T}} }}, $$

where *k*_T_ signified the turbulence energy; *ε*_T_ was the dissipation rate of the turbulence energy; *P*_T_ meant the parameter related to the velocity gradient; *G*_*T*_ denoted the parameter related to buoyancy; and *C*_1_ = 1.44, *C*_2_ = 1.92, *C*_3_ = 0.2.

The VOF method was applied to track the free water surface (the interface between water and air). After considering the area fraction and volume fraction, the VOF equation was as follows [27]:

7 $$ \frac{\partial \alpha }{\partial t} + \frac{1}{{V_{F} }}\left[ {\frac{\partial }{\partial x}\left( {\alpha A_{x} u_{x} } \right) + \frac{\partial }{\partial y}\left( {\alpha A_{y} u_{y} } \right) + \frac{\partial }{\partial z}\left( {\alpha A_{z} u_{z} } \right)} \right] = 0, $$

where *α* denoted the volume fraction ( = 1 meant the cell was full of water).

### CFD model design and mesh generation

**Fig. 2:**
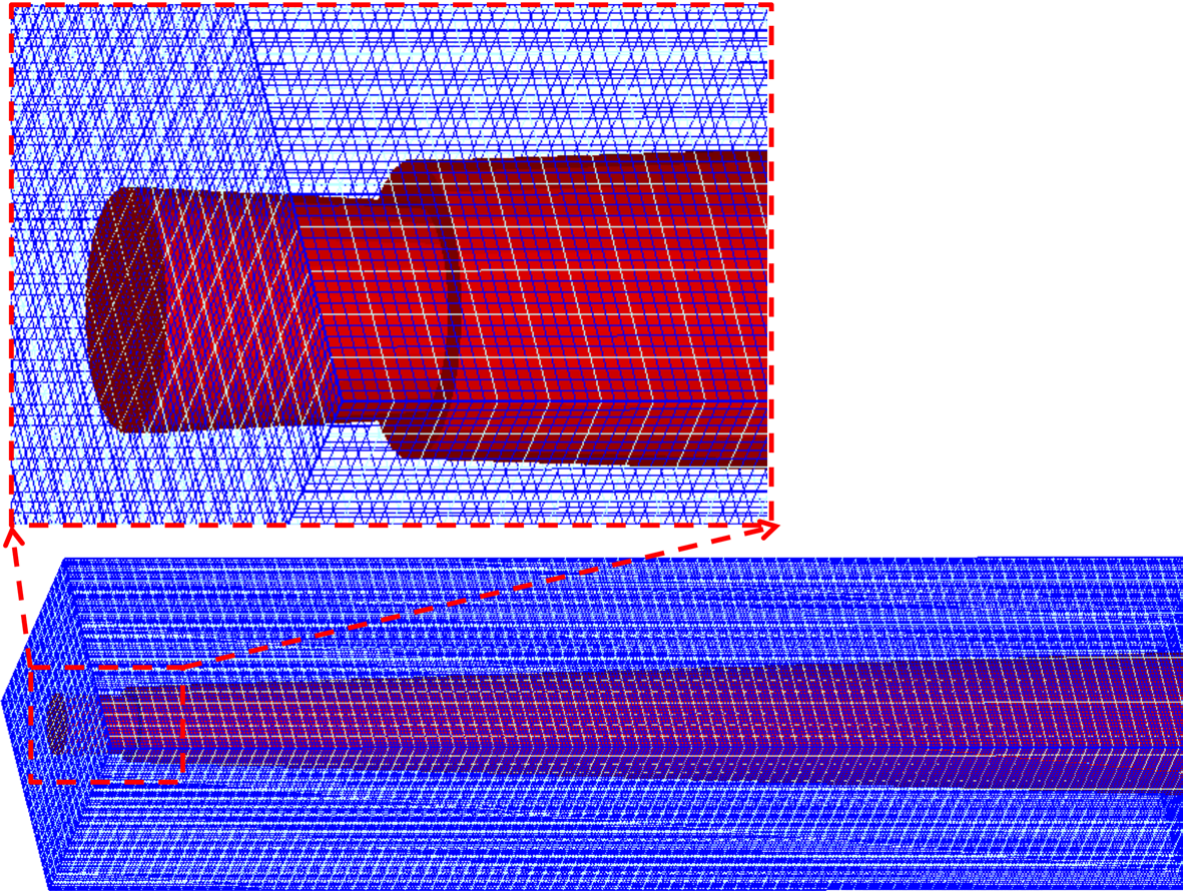
The overview of the mesh density of the CFD model in the apical part of the root canal

The three-dimensional CAD canal and needle geometrical model was created and imported into the Flow-3D codes (Flow Science Inc., USA). The FAVOR technique was applied in the process of mesh generation and the computational grids was 0.02 mm, with a total of 1,647,300 hexahedral elements (Fig. 2). The fluid was ejected into the canal from the outlet of the needle and flew out through the orifice of the root canal where atmospheric pressure was imposed. The canal and needle were considered to be fully filled with irrigant. Gravity factor was directed at the direction of the negative *y*-axis.

The mass source was used to simulate the entrance of the needle lumen into the root canal and by changing the displacement of the mass source, the movement of the needle in clinical practice could be modeled. One needle was kept stationary at 3.5 mm from the apical foramen during the whole irrigation process (Case_0_). Three needles were kept in motion with amplitudes of 1, 2, 3 mm at a frequency of 1 Hz (Case_A1_, Case_A2_, Case_A3_), which meant the needles were in constant 2, 4, 6 mm up-and-down motions every second, respectively. Another three needles were set to be moving up and down in 4 mm motions with different frequencies. To be specific, three frequencies (*F* = 1, 2, 3) were adopted, which meant the needle made 1,2,3 up-and-down motions in one second, respectively (Case_F1_, Case_F2_, Case_F3_). Moreover, the movement locus of the needle was achieved by firstly moving away from the apex and then approaching to the starting point (3.5 mm from the apical foramen) in constant motions.

### Boundary conditions and numeric simulation

No-slip boundary condition was adopted to the walls of the root canal and of the needle with the assumptions that the walls were rigid, smooth, and impermeable. The walls of the root canal were defined as wall boundary condition, while other boundaries were defined as pressure boundary condition, which meant the relative pressure was zero. (Moved to “The Geometry construction for a CFD irrigation model” section.)

When solving the governing equations, in order to get stable numerical solution, the time step (∆*t*) must be less than or at most equal to ∆*x*/*c* based on the definition of Courant number (∆*x* was the cell size in the direction of the velocity, *c* was the magnitude of the velocity through the cell) [28]. In this study, ∆*x* was 2 × 10^−5^ m and *c* was 8.6 m/s. Therefore, the minimum time step for this study was 2.3 × 10^−6^ s, however, in order to improve the computational accuracy, a smaller time step of 1 × 10^−7^ s was used throughout the whole calculations. In addition, the computational time for each case was set to 10 s. When the flow developed fully and the parameters of interest reached a steady state, 1 s was adopted in order to allow one period (*T*) for the cases kept in motion. Computations were carried out on a Windows 7 Computer workstation with 2 CPU and 128 G RAM. A series of computations were performed where the needle was kept stationary or moved up and down in motions. The flow fields computed were compared including flow pattern, velocity magnitude, and apical pressure.

### Statistical analysis

Statistical analysis was analyzed with statistical software (IBM SPSS Statistics, v22.0; IBM Corp). 1-way analysis of variance (ANOVA) was performed to identify statistically significant difference among different groups ( = 0.05).

## Results

### Model validation

First of all, the 3D CFD model was validated by comparing with previous numerical results [14, 15]. The mean apical pressure of the stationary case (18.04 kPa) (Table 1) generated in the present study agreed well with the mean apical pressure of previous CFD analysis using the same canal model and irrigation speed [14, 15]. Differences with other CFD studies might be attributed to different experimental settings and turbulence models [16]. Therefore, the validity of the current model could be verified.

**Table 1:** Mean flow velocity and apical pressure of different cases

Cases	*V*_*m*_ (m/s)	*P*_0.1_ (%)_1_	*P*_*m*_ (kPa)
Case_0_	3.84	95	18.04
Case_A1_	2.73	86	15.13
Case_A2_	1.48	87	13.41
Case_A3_	0.97	69	11.65
Case_F1_	1.48	87	13.41
Case_F2_	1.58	88	14.33
Case_F2_	1.80	88	13.75

*V*_*m*_, mean *y*-velocity magnitude (m/s) at point A; *P*_0.1_, percentage of time when the *y*-velocity magnitude at point A is higher than 0.1 m/s; *P*_*m*_, mean apical pressure (kPa)

### Comparison of the stationary needle and the needles in motions

**Fig. 3:**
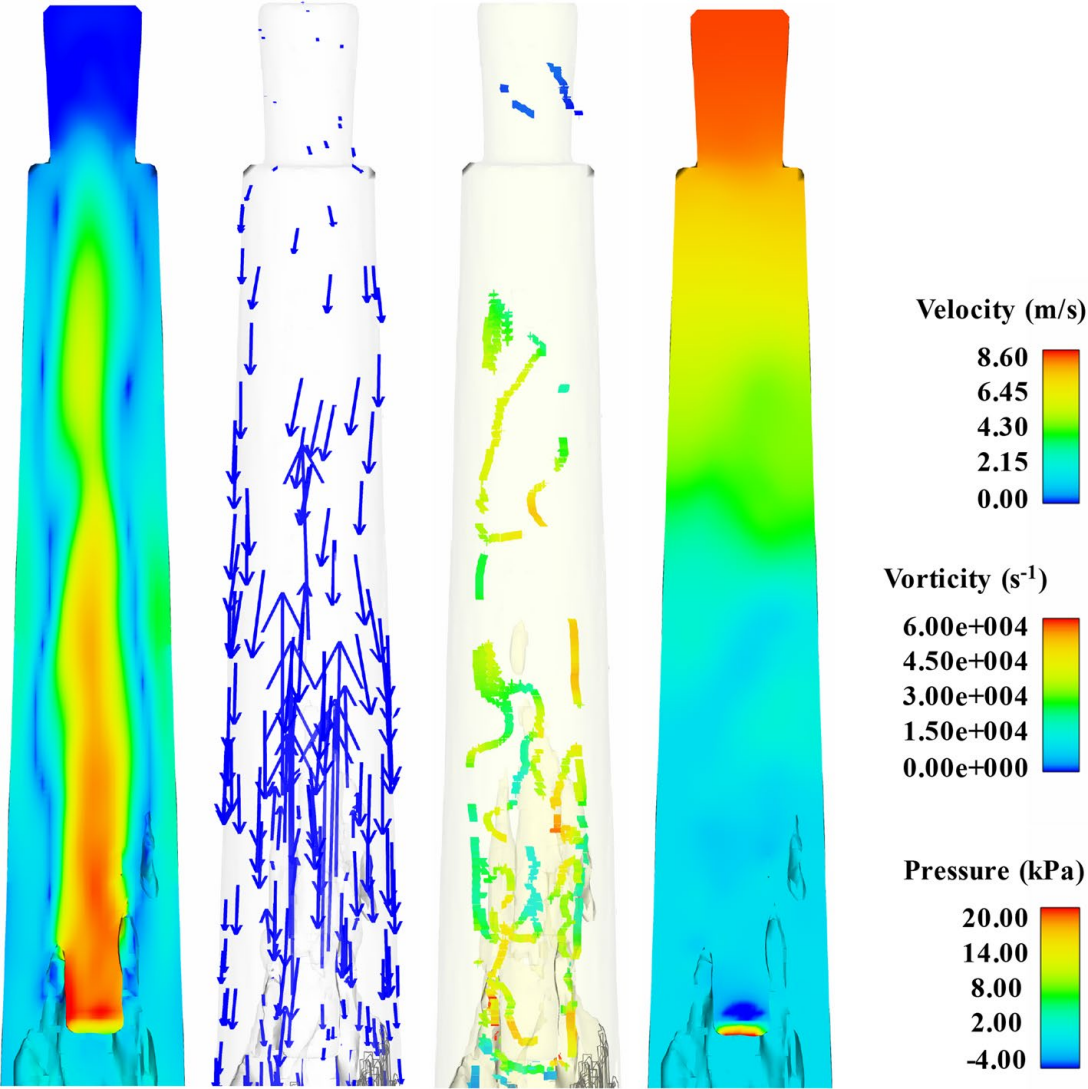
The distribution of the velocity field, velocity vector, vorticity, and pressure field of Case_0_ at 1/2*T* in the apical part of the root canal

**Fig. 4:**
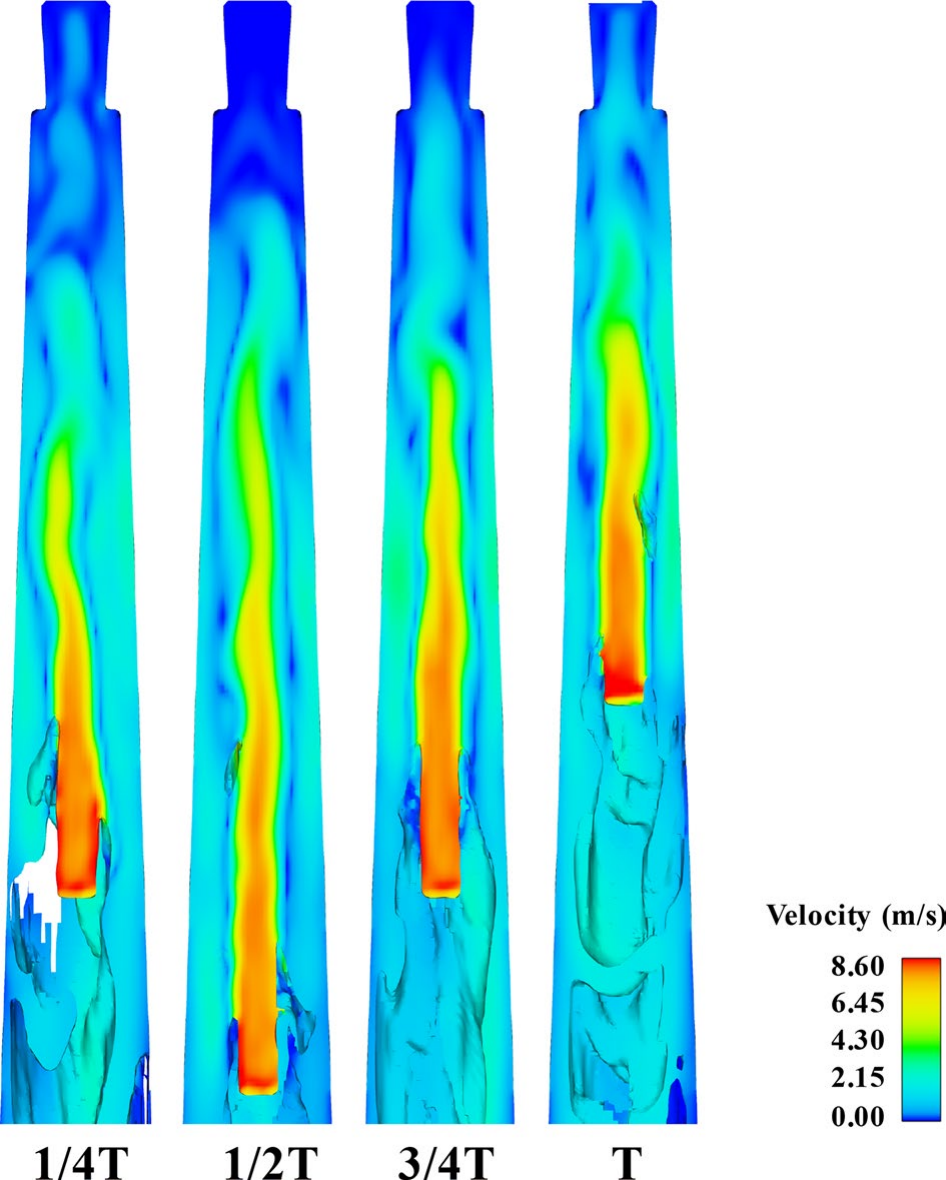
The distribution of the velocity field of Case_A1_ at 1/4*T*, 1/2*T*, 3/4*T,* and *T* within one period (*T*) in the apical part of the root canal

**Fig. 5:**
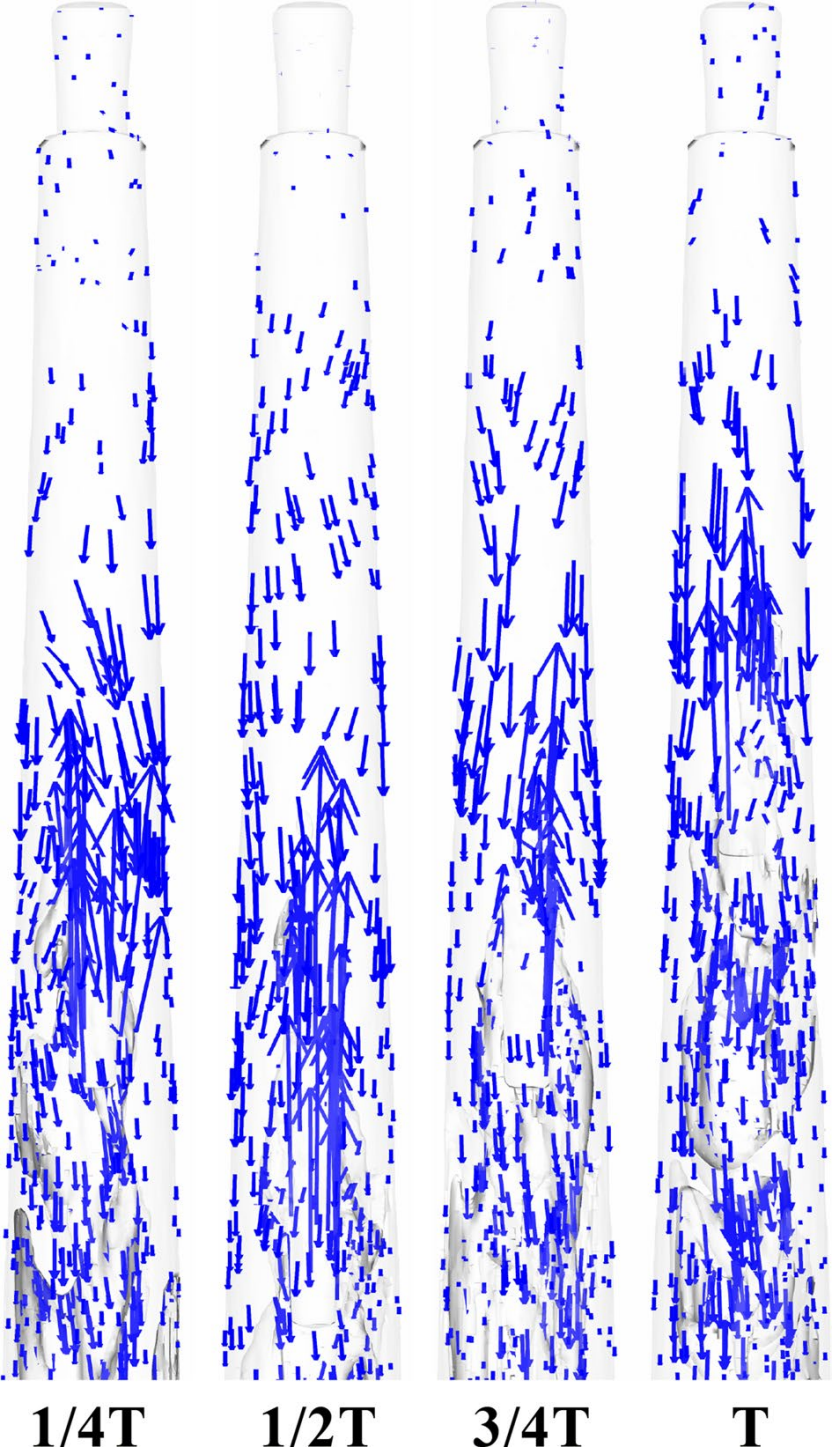
The distribution of the velocity vector of Case_A1_ at 1/4*T*, 1/2*T*, 3/4*T*, and *T* within one period (*T*) in the apical part of the root canal

**Fig. 6:**
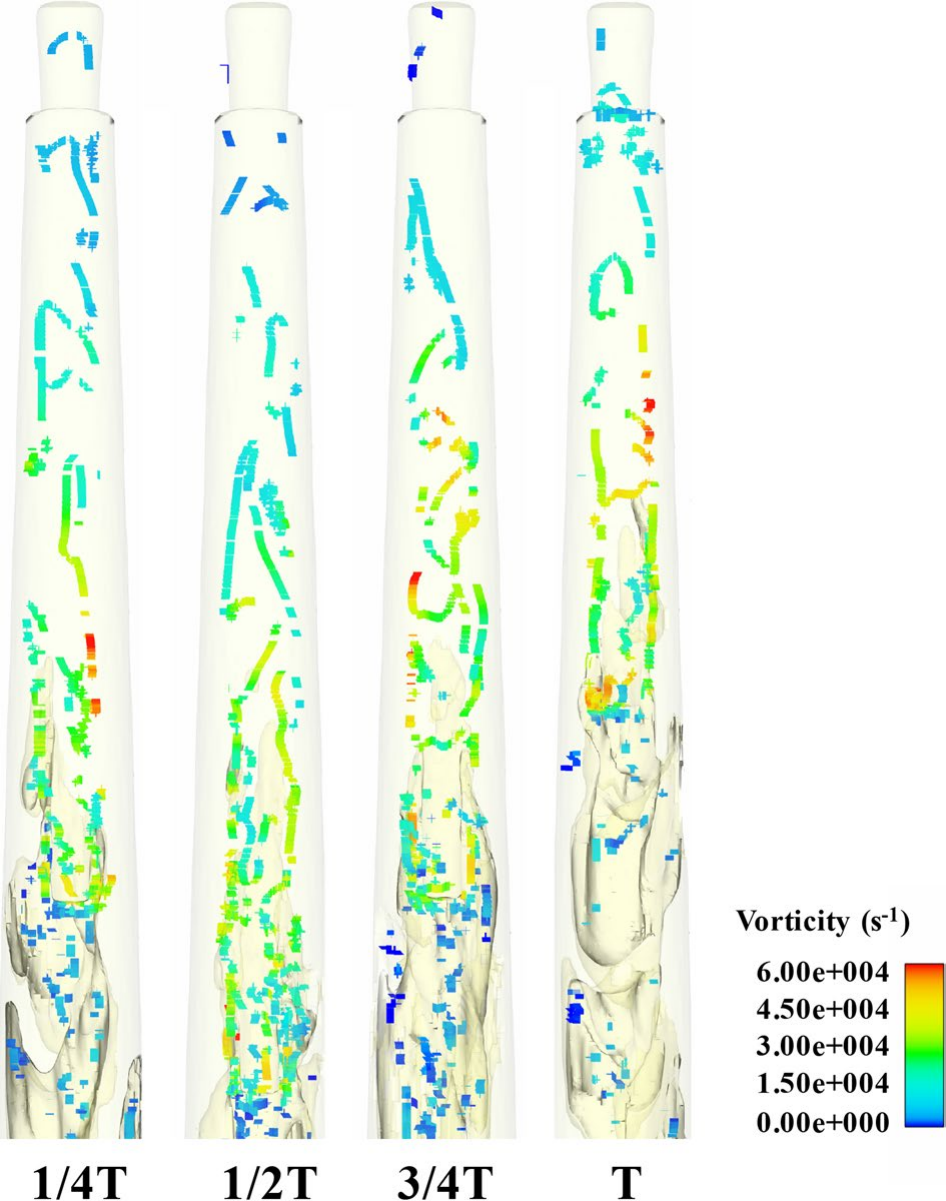
The distribution of the vorticity of Case_A1_ at 1/4*T*, 1/2*T*, 3/4*T*, and *T* within one period (*T*) in the apical part of the root canal

**Fig. 7:**
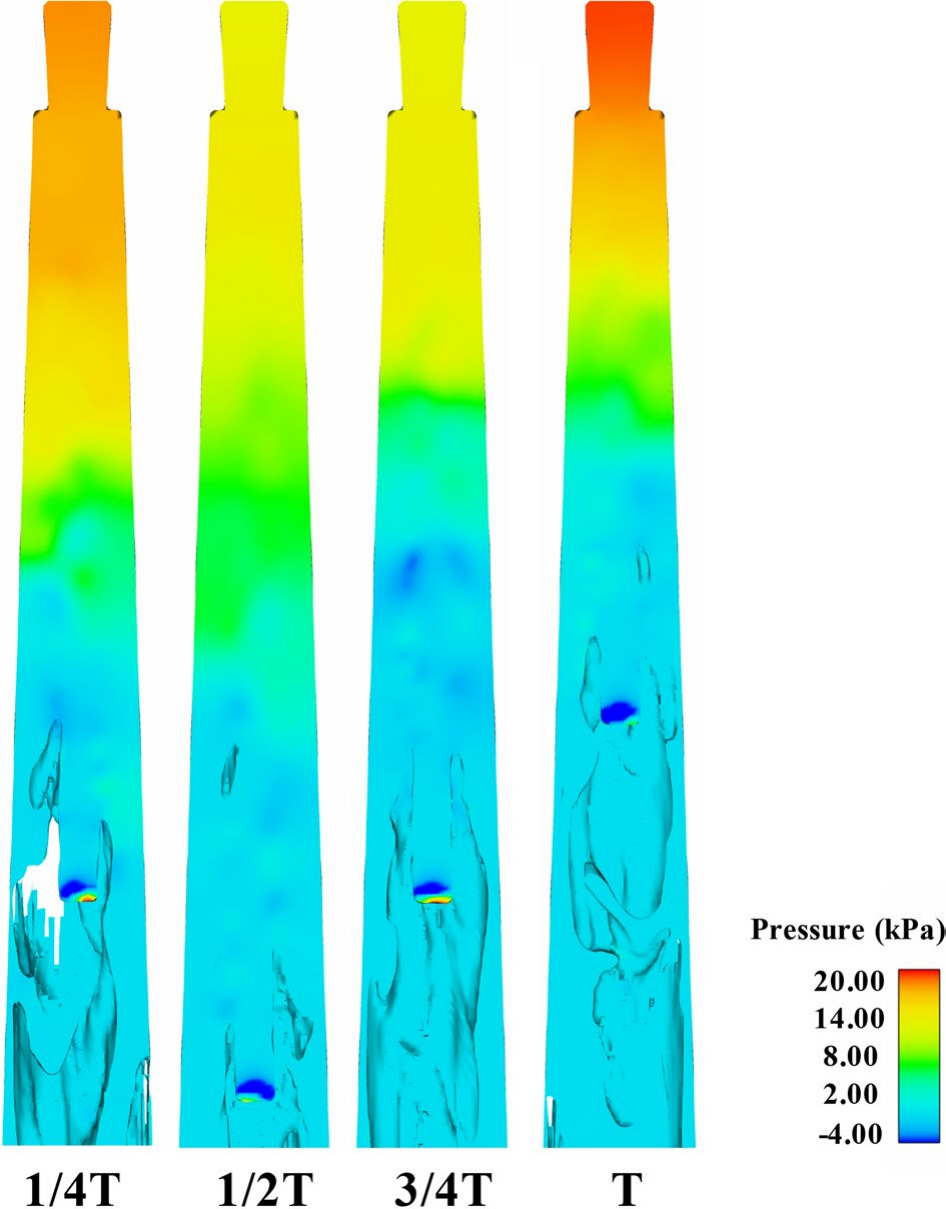
The distribution of the pressure field of Case_A1_ at 1/4*T*, 1/2*T*, 3/4*T*, and *T* within one period (*T*) in the apical part of the root canal

The distribution of velocity field, velocity vector, vorticity, and pressure field at 1/2 *T* (*T* = one period) when the needle remained static (Case_0_) is presented in Fig. 3. In addition, the distribution of the velocity field (Fig. 4), velocity vector (Fig. 5), vorticity (Fig. 6), and pressure field (Fig. 7) of Case_A1_ at certain time: 1/4 T, 1/2 T, 3/4 T, and T in the apical part of the root canal is also shown and they provide a detailed overview of the variations of fluid field of Case_A1_. As shown, the value of flow parameters of Case_A1_ gradually decreased as the needle moved away from the apical foramen, while increased when the needle approached the apex. In addition, the variations of velocity and pressure field of Case_0_ and Case_A1_ within a second were also demonstrated by one video (Additional file 1: Video S1).

**Fig. 8:**
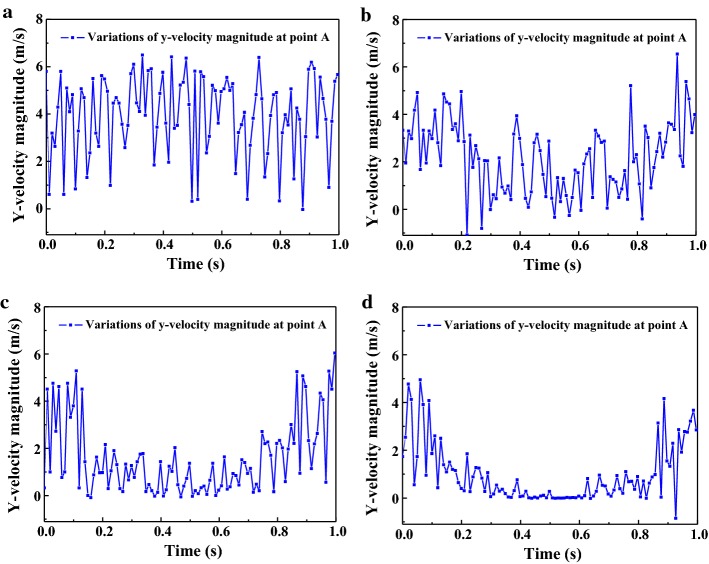
The variations the axial *y*-component of irrigant velocity at point A of Case_0_ (**a**), Case_A1_ (**b**), Case_A2_ (**c**), and Case_A3_ (**d**) in one second

**Fig. 9:**
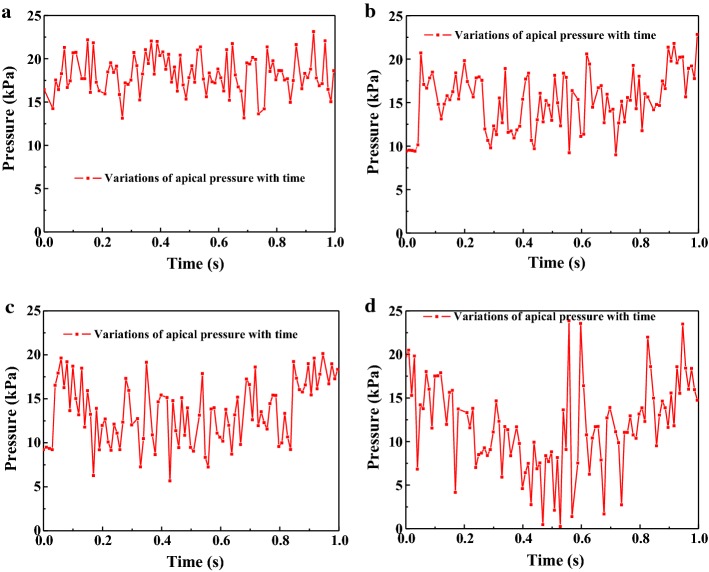
The variations of apical pressure of Case_0_ (**a**), Case_A1_ (**b**), Case_A2_ (**c**), and Case_A3_ (**d**) in one second

The variations of *y*-velocity at point A and apical pressure of the stationary needle (Case_0_) and needle in 2 mm up-and-down motions (Case_A1_) within a second are presented in Figs. 8a, b and 9a, b. Like the fluid field developed within the canal (Figs. 3, 4, 5, 6, 7), the distinction between the stationary case and Case_A1_ was still eminent. To be specific, Case_0_ exhibited relatively higher irrigant velocity and apical pressure all the time, while Case_A1_ gained smaller irrigant velocity and apical pressure, especially when the needle moved away from the apex. In addition, Table 1 shows that the mean *y*-velocity magnitude at point A, mean apical pressure, and the percentage of time when the *y*-velocity magnitude at point A is higher than 0.1 m/s (*P*_0.1_) of Case_0_ were all larger than that of Case_A1_.

### Comparison of the needles in motions with different amplitudes

To investigate the influence of the amplitude on irrigation efficiency, various amplitudes of 1, 2, 3 mm (Case_A1_, Case_A2_, and Case_A3_) were adopted. Figures 8, 9 show the variations of *y*-velocity at point A and apical pressure of needles with different amplitudes. Furthermore, the *V*_*m*_, *P*_*m*_, and *P*_0.1_ of Case_A1_, Case_A2_, and Case_A3_ are summarized in Table 1. As shown, the flow velocity and apical pressure in different cases presented a similar trend, that is, the lager the amplitude of the needle, the more unstable and smaller the flow velocity and apical pressure. However, when comparing *P*_0.1_ of different amplitudes, Case_A1_ is similar to Case_A2_ (86% and 87%), while Case_A3_ gained smaller *P*_0.1_ (69%).

### Comparison of the needles in motions with different frequencies

**Fig. 10:**
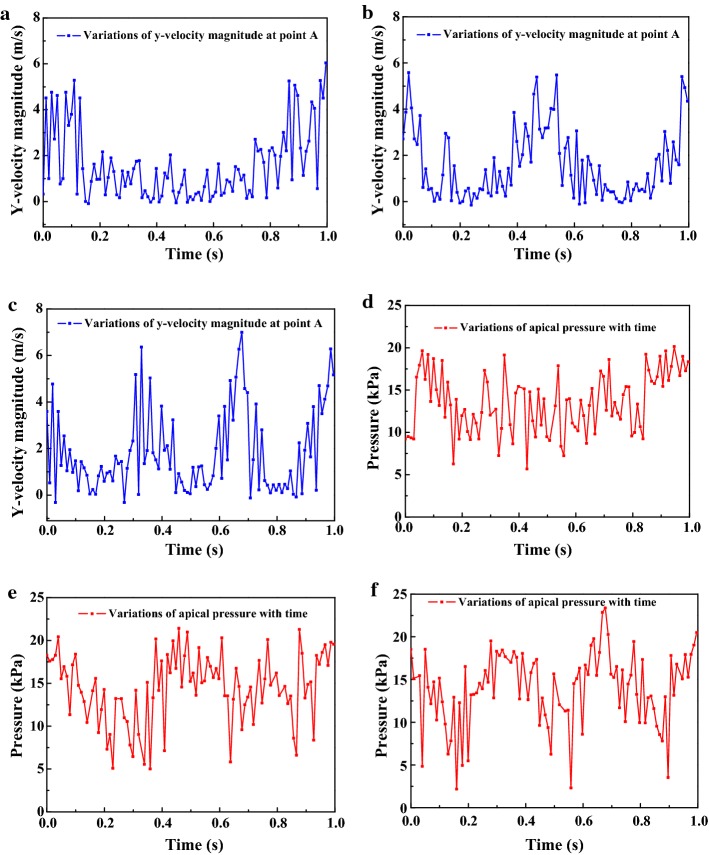
The variations of the axial *y*-component of irrigant velocity at point A and apical pressure of Case _F1_ (**a**, **d**), Case_F2_ (**b**, **e**), and Case _F3_ (**c**, **f**) in one second

The effect of frequency on irrigation efficiency was also studied and the variations of *y*-velocity at point A and apical pressure of needles with different frequencies (*F* = 1, 2, 3, Case_F1_, Case_F2_, and Case_F3_) are presented in Fig. 10. The magnitude of flow velocity and apical pressure in different cases underwent periodic alterations and were inconsistent with the needle movement locus. Moreover, the *V*_*m*_, *P*_*m*_, and *P*_0.1_ are also shown in Table 1 and the results show that there were no significant differences among Case_F1_, Case_F2_, and Case_F3_ in the magnitude of mean flow velocity (1.48, 1.58, 1.80), *P*_0.1_ (87%, 88%, 88%), and apical pressure (13.41, 14.33, 13.75) (*P *> 0.05).

## Discussion

CFD simulations have previously shown clinical relevance in a range of studies [13–17], but without incorporating the motion of the needles. This study described the first simulation to investigate the influence of needle movement on the root canal irrigation using the CFD model. The dynamic alteration of the flow characteristics such as flow velocity and pressure were examined when the needle was kept stationary or moved up and down in constant motions with different amplitudes and frequencies.

The stationary case (Case0) presented relative high flow velocity all the time, while the cases in motions underwent evident periodic alterations of velocity with much lower mean flow velocity. Since it has been pointed out by previous studies that the distribution of *y*-velocity along the longitudinal axis of the root canal (*y*-axis) acts as a vital role in determining the irrigant replacement and the flush effect [13–17] and velocities higher than 0.1 m/s were considered clinically significant for adequate irrigant replacement [14, 15], it could be speculated that the stationary case would achieve improved irrigation replacement, as depicted by *P*_0.1_ (the percentage of time when the *y*-velocity magnitude at point A is higher than 0.1 m/s) (Table 1). However, besides irrigation replacement, other factors should also be taken into consideration when assessing an irrigation protocol. For example, a chief complication during irrigation is the extrusion of irrigant into periapical tissues, leading to severe pain and tissue destruction [3, 12]. If the pressure at the apical foramen exceeds the backpressure of the periradicular tissue, periapical extrusion would probably happen and complications caused by cytotoxic effects of irrigant might occur [29]. As stated before, the stationary case gained much higher pressure all through the irrigation process, hence it is more likely to force the irrigant and debris into the periapical tissue. In comparison, the needles in motions presented a distinct decrease in pressure when the needle moved away from the apex and thus gained much lower apical pressure. Such trend would pose much lower risks of injury to the periradicular tissue. Moreover, cases in motions with smaller amplitude like Case_A1_ and Case_A2_ could also gain adequate irrigant replacement (86% and 87%, respectively). Therefore, it is reasonable to assume that the needles in motions would produce safer irrigation effect yet adequate irrigant replacement if moving the needle up and down with smaller amplitude.

When comparing cases in motions with different amplitudes, the overall variation trend of the flow velocity and apical pressure was similar and in parallel with the periodic motion of the needles. As shown, the irrigant replacement was not sacrificed when increasing the amplitude from 1 mm (Case_A1_, 86%) to 2 mm (Case_A2_, 87%). However, when the amplitude was increased to 3 mm in Case_A3_, the irrigant replacement became less efficient (69%) (Table 1). Therefore, it could be estimated that the irrigant replacement would be insufficient if the moving amplitude is larger than 3 mm. On the other hand, with the decrease of the moving amplitude, the apical pressure, as well as the risk of periapical extrusion would be higher [14–17]. Therefore, increasing the moving amplitude would be safer during irrigation. However, because there is no definite evidence on the minimum irrigant pressure that leads to extrusion, the risk of apical extrusion can only be estimated by comparison between different cases. Therefore, a reasonable compromise would be to move the needle with a moderate amplitude to get a relatively satisfactory flush effect and lower apical pressure meanwhile during irrigation.

Furthermore, a noteworthy finding of this study was the influence of the moving frequencies of the needle on irrigation efficiency. As indicated by the results, no significant differences were observed among different cases in the magnitude of mean flow velocity, apical pressure, and the percentage of clinically efficient velocities (*P*_0.1_). Though the mean flow velocity is a little higher when increasing the frequencies, the *P*_0.1_ and mean apical pressure in the three cases were almost the same, indicating similar irrigant replacement and risk of apical extrusion. Hence, the moving frequencies of the needles were not highly related to the irrigation efficiency and thus might not be the main concern during irrigation process. Therefore, it would be unnecessary to move the needles very fast or very slow to improve irrigation efficiency and should be just the practitioners’ personal preference in terms of motion frequency.

Nevertheless, the findings of this study should further be evaluated before any clinical suggestion is made. First of all, it is hard to say exactly which amplitude should be employed since there are no safety limits set for the apical wall pressure so far and many other additional factors such as the needle depth, needle type, and the apical foramen size and taper of canal would also have great impacts on the irrigation outcome. In addition, there are some limitations of the model applied. For example, the geometry of the root canal was simplified in order to allow comparison with previous literature [14, 15]. The walls of the root canals were assumed to be smooth and impermeable and this assumption may not be clinically relevant. Moreover, the gravity would vary according to the canal and how the patient sits on the chair was not taken into consideration in this study. Therefore, further studies should be conducted to provide a more comprehensive guidance to clinical practice.

## Conclusion

The present study presented an initial attempt to use a CFD model to investigate the influence of the needle movement on the root canal irrigation. Within the limitations of the study, this numerical model provides some basic understanding of the effect of needle movement on the flow velocity and apical pressure that experimental measurements are difficult to perform. First of all, the stationary case gained higher irrigant replacement yet would also pose much higher risk of injury to the periradicular tissue. Therefore, the fear of irrigant extrusion has led to the suggestion of keeping the needle in motion while manually irrigating the canal. In addition, comparison of different cases in motions demonstrated that a needle moving up and down with a moderate amplitude during manual root canal irrigation would result in better flush effect while reducing the risk of periapical extrusion; and the motion frequency was not highly relevant in terms of the irrigation efficiency and should be practitioners’ personal preferences.

## Additional file

**Additional file 1.** Additional video.

## Data Availability

The datasets used and analyzed during the current study are available from the corresponding author on reasonable request.
